# Supplementary material for: Mechanisms governing the pioneering and redistribution capabilities of the non-classical pioneer PU.1
Source: Nat Commun. 2020 Jan 21;11:402. doi: 10.1038/s41467-019-13960-2 (PMC6972792; doi:10.1038/s41467-019-13960-2)
Supplement: Supplementary file 7 — Source data [file 41467_2019_13960_MOESM7_ESM.zip › Source_Data/FigureS2/FigureS2B_MotifScanOutput/KmeansCluster14/homerResults/motif2.similar.html]

motif2

## Information for motif2

C
T
G
A
T
C
A
G
A
G
T
C
A
G
T
C
C
G
T
A
G
T
C
A
G
A
C
T
T
A
G
C
T
C
G
A
T
C
A
G
T
G
C
A
C
T
A
G
  
Reverse Opposite:  

G
A
T
C
A
C
G
T
A
G
T
C
A
G
C
T
A
T
C
G
C
T
G
A
A
C
G
T
C
G
A
T
C
T
A
G
A
C
T
G
A
G
T
C
G
A
C
T
  

|  |  |
| --- | --- |
| p-value: | 1e-65 |
| log p-value: | -1.517e+02 |
| Information Content per bp: | 1.613 |
| Number of Target Sequences with motif | 133.0 |
| Percentage of Target Sequences with motif | 26.08% |
| Number of Background Sequences with motif | 1789.5 |
| Percentage of Background Sequences with motif | 3.95% |
| Average Position of motif in Targets | 104.5 +/- 56.4bp |
| Average Position of motif in Background | 93.2 +/- 65.5bp |
| Strand Bias (log2 ratio + to - strand density) | 0.1 |
| Multiplicity (# of sites on avg that occur together) | 1.28 |
| Motif File: | file (matrix) reverse opposite |

### Similar de novo motifs found

|  |  |  |  |  |  |  |  |
| --- | --- | --- | --- | --- | --- | --- | --- |
| Rank | Match Score | Redundant Motif | P-value | log P-value | % of Targets | % of Background | Motif file |
| 1 | 0.972 | A G T C C T G A T C A G A G T C A G T C C G T A C G T A A C G T T G A C T C G A C A T G T C G A C T A G A T C G | 1e-64 | -149.209600 | 24.12% | 3.33% | motif file (matrix) |
| 2 | 0.979 | A G T C T C G A T C A G A G T C A T G C C G T A C T G A A G C T T A G C T C G A T A C G T C G A C T A G | 1e-61 | -140.929506 | 28.43% | 5.32% | motif file (matrix) |
| 3 | 0.923 | C T A G A G T C A G T C C G T A C G T A C G A T T A C G C T A G T C A G C T G A C A G T | 1e-60 | -138.309581 | 24.71% | 3.92% | motif file (matrix) |
| 4 | 0.942 | C T A G A G T C A G T C C G T A C G T A G C A T T A G C C T G A C T A G T C A G | 1e-58 | -135.313253 | 27.25% | 5.07% | motif file (matrix) |
| 5 | 0.933 | A G T C A C T G C T G A C G A T G C A T C A T G C T A G A G T C | 1e-57 | -132.542275 | 29.22% | 6.07% | motif file (matrix) |
| 6 | 0.929 | A G T C A T C G C T G A C G A T C G A T C A T G A C T G A G T C A G T C | 1e-54 | -124.857802 | 29.41% | 6.56% | motif file (matrix) |
| 7 | 0.927 | C G T A A C G T A C G T A C T G A C T G A G T C A G C T | 1e-48 | -111.568614 | 33.33% | 9.43% | motif file (matrix) |
| 8 | 0.875 | A T G C C G T A A C T G A G T C A G T C C G T A T C G A A G C T A T G C C T A G A G T C | 1e-36 | -84.308106 | 13.53% | 1.78% | motif file (matrix) |
| 9 | 0.849 | A G T C T A G C C T G A C G T A G C A T G T A C C T G A T C A G G C T A | 1e-34 | -80.465587 | 14.51% | 2.25% | motif file (matrix) |
| 10 | 0.779 | C G T A G T A C T A G C G T C A C G T A G C A T G T A C G T C A A T C G T G C A T C A G T C G A | 1e-25 | -59.465772 | 10.59% | 1.61% | motif file (matrix) |
| 11 | 0.753 | A G T C G A T C G T C A T G C A G C A T A T G C T G C A T A C G C G T A T C A G | 1e-23 | -54.032200 | 19.02% | 5.85% | motif file (matrix) |
